# Determinants of the managerial staff’s disposition towards e-payment platforms in public tertiary hospitals in Enugu, Nigeria: a cross-sectional study

**DOI:** 10.1186/s12913-023-10302-3

**Published:** 2023-11-11

**Authors:** James Okechukwu Abugu, Amaechi Marcellus Chukwu, Ogochukwu Kelechi Onyeso, Chiedozie James Alumona, Israel Ikechukwu Adandom, Ogo-Amaechi D. Chukwu, Oluwagbohunmi A. Awosoga

**Affiliations:** 1 Department of Marketing, Faculty of Business Administration, University of Nigeria, Nsukka, Enugu Nigeria; 2 Department of Medical Rehabilitation, Faculty of Health Sciences and Technology, College of Medicine, University of Nigeria, Nsukka, Enugu Nigeria; 3 Faculty of Health Sciences, University of Lethbridge, Lethbridge, AB Canada; 4 Department of Kinesiology, University of Alabama, Tuscaloosa, AL USA; 5 Department of Computer Science, Faculty of Physical Sciences, College of Medicine, University of Nigeria, Nsukka, Enugu Nigeria

**Keywords:** Health financing, Health services, Health expenditure, Mobile money, Perception

## Abstract

**Background:**

Many Nigerians pay out-of-pocket for their health care, and some hospitals have started utilising e-payment systems to increase transactional efficiency. The study investigated the type and usage of e-payment platforms in public hospitals and the factors that may influence the managerial staff’s disposition towards using the e-payment system.

**Methods:**

We conducted a cross-sectional survey of 300 managerial staff within the four public tertiary hospitals in Enugu, Nigeria, through proportionate quota sampling. The survey obtained participants’ demographic characteristics, types of e-payment platforms, managerial staff’s technophobia, perception of credibility, and disposition towards e-payment. Data were analysed using descriptive statistics, Spearman correlation, and hierarchical linear regression.

**Results:**

The majority of the respondents (n = 278, 92.7% completion rate) aged 43.4 ± 7.6 years were females (59.0%) with a bachelor’s degree (54.7%). Their disposition (80.0%±17.9%), perceptions of the usefulness (85.7 ± 13.9%), and user-friendliness (80.5 ± 18.1%) of e-payment in the hospital were positive, credibility (72.6 ± 20.1%) and technophobia (68.0 ± 20.7%) were moderate. There was a negative correlation between technophobia and disposition toward the use of e-payment (*ρ* = -0.50, *P* < 0.001). Significant multivariate predictors of managerial disposition towards e-payment were; being a woman (β = 0.12, *P* = 0.033), married (β = 0.18, *P* = 0.003), positive perception of usefulness (β = 0.14, *P* = 0.025), and credibility (β = 0.15, *P* = 0.032).

**Conclusion:**

Most participants had a positive disposition towards e-payment in public hospitals. However, managers with technophobia, a negative perception of e-payment usefulness, and credibility had a lesser disposition to its use. To ensure the universal implementation of e-payment in Nigerian hospitals, the service providers should make the e-payment platforms more secure and user-friendly to health services consumers and providers.

## Background

Nigeria is a middle-low-income sub-Saharan African country with an estimated population of over 200 million people [1] and an annual population growth rate of 2.41% [2]. Nigeria is the most populous country in Africa and the seventh in the world [1]. Sub-Saharan African countries continue to be plagued by infectious and non-communicable diseases, leading to high demand for health services [3]. On the contrary, the Nigerian healthcare sector is facing complex challenges, including poor welfare and brain-drain of health care workers, infrastructural decay, leadership tussle, poor policy design and implementation, and paucity of health insurance coverage at the federal and state levels [4, 5]. More than 96% of Nigerians not covered by the National Health Insurance Scheme (NHIS) pay out-of-pocket for health services [6]. This transactional form of health expenditure is characterised by on-spot billing and payment before health services can be rendered [7]. Before the advent of technology-driven e-banking in Nigeria, payment for these services was by cash or cheques deposited physically at the hospital’s pay points or designated bank branches within or outside the hospital.

E-payment is a way of transferring funds between bank accounts or paying bills through online real-time computerised systems [8] without the use of physical cheques or cash [9]. In Nigeria, e-payment platforms such as automated teller machines (ATMs), unstructured supplementary service data, online and mobile banking Apps., point-of-sale machines, and other portable e-payment systems have been introduced to ease financial transactions [10, 11]. Though the ATMs were primarily designed to facilitate cash withdrawal, they are valuable means of electronic cash transfer for people without mobile banking installed smart devices [12]. The introduction of the cashless policy by the Federal Government of Nigeria in 2012 led to the adoption of e-payment by public agencies [13]. However, some traditional bureaucratic institutions, such as publicly run hospitals, have been slow in adjusting their systems to conform with e-payment.

Health service consumers will benefit immensely from the full implementation of an e-payment system within public hospitals in Nigeria. It will reduce patient waiting time in clinics, physical stress for caregivers, and time lost due to queuing for cash transactions at pay points [14]. The outdated cash, teller, and cheque systems slowed down transaction settlements because of overreliance on human operators and manual processes [12]. Sometimes, evidence of payment must be processed, posted, and verified with the hospital ledger before the commencement of treatment [7]. In contrast, e-banking automates banking services, allowing patients and the hospital management to access their accounts and receipts of payment from portable electronic devices such as laptops and smartphones [11]. Aside from clients’ ease of payment, the service providers also benefit from e-payment systems. The system can run 24 h alongside clinical services without human intervention, reducing the cost of off-peak hour and night duty staff, human fatigue, error, and financial dishonesty [7, 15]. Though the benefits of e-payment in Nigeria appear to outweigh its limitations, there are still concerns about electricity, internet services, network interruption, and internet fraud [16]. All these may lead to e-payment technophobia among some managerial staff of public hospitals.

This study was grounded in Fred Davis’s technology acceptance model (TAM) of 1989, which presumes a mediating role of *perceived ease of use* and *perceived usefulness* in a complex relationship between system characteristics and potential system usage [17, 18]. TAM is a dominant model in explaining users’ disposition toward technology [17, 18]. We conceptualised (managerial staff’s) perceptions of usefulness, ease of use [18], and system characteristics such as credibility and security [19] as determinants of disposition to institutional use of e-payment platforms.

Using e-payment systems could lead to better outcomes for patients by enhancing the performance of the healthcare system and mitigating transactional stress among patients, caregivers, and hospital staff [20–22]. However, there is a paucity of research on the policymakers’ perceptions and acceptability of e-payment among public institutions in Nigeria. Therefore, this study investigated the availability of e-payment platforms and the managerial staff’s disposition towards using e-payment systems in Nigerian hospitals. We hypothesised that there would be no significant (1) correlations among the number of hospital’s e-payment platforms, managerial staff disposition, technophobia, perceived usefulness, ease of use, and credibility, and (2) a set of factors that could predict managerial staff disposition towards the use of e-payment in the hospitals.

## Methods

### Study design

The study was a cross-sectional survey. Ethical clearance was obtained from the Health Research Ethics Committee of the Molecular Pathology Institute, Enugu State, Nigeria (MPIHREC/2023/05/01/B/0005). Individual written informed consent was obtained from the participants, who were duly informed of the study protocol and their rights to withdraw at any point in the study without any consequence. Participants’ anonymity, privacy, confidentiality, and other guidelines from the Helsinki Declaration on research involving human subjects were strictly adhered to. The study was reported following the Strengthening the Reporting of Observational Studies in Epidemiology (STROBE) guidelines for reporting cross-sectional studies [23].

### Setting

The study was conducted in public tertiary hospitals in Enugu, Nigeria. They include University of Nigeria Teaching Hospital (UNTH), Enugu State University Teaching Hospital (ESUTH), National Orthopaedic Hospital, Enugu (NOHE), and Federal Neuropsychiatric Hospital, Enugu (FNHE), which serve as referral and specialist centres for the southeastern region of Nigeria. The hospitals’ numerous staff and large patient turnouts attract financial activities within the premises, such as payment for hospital services, food vendors, and medical supply outlets.

### Participants and eligibility

The participants were managerial staff of the tertiary hospitals in Enugu. Information obtained from the hospital’s human resource units showed the managerial staff strength: UNTH = 3,500, ESUTH = 1100, NOHE = 400, and FNHE = 300. The eligibility criteria were being an employed hospital staff with at least two years of work experience on a managerial cadre (grade level 14 and above) in any department and willingness to sign the informed consent and complete the questionnaire. Participants were excluded if they were more than 60 years old or had a cognitive problem.

### Sample size determination

A priori power analysis was performed using G*Power 3.1.9.4 software based on a power of 0.95, an alpha level of 0.05, a moderate effect size of 0.1, and 9 predictors under the linear regression fixed model. The minimum calculated sample size was 245. However, 300 participants were recruited in anticipation of a 15% incomplete questionnaire.

### Sampling technique and bias

Sampling bias was minimised by (1) sampling all four publicly funded tertiary hospitals in Enugu, (2) employing proportionate sampling across the hospitals, and (3) employing a consecutive sampling in the recruitment of individual participants who met the eligibility criteria within the hospitals. The proportionate quota sampling technique ensured that none of the hospitals was underrepresented. The calculation gave UNTH = 195, ESUTH = 63, NOHE = 24, FNHE = 18, and total = 300.

### Research instrument

The survey instrument was a 35-item questionnaire comprising seven sections. Section A (1 item) asked about the types of e-payment platforms in the hospital. Sections B (5 items), C (5 items), D (5 items), E (5 items) and F (10 items) collected information on the participants’ perceived usefulness, ease of use, credibility, technophobia, and disposition towards the use of e-payment in the hospital, respectively. Participants’ sociodemographic characteristics were collected in section G (4 items). The questionnaire was drafted by three experts in questionnaire development and questionnaire-based research following an in-depth literature review. A four-member panel comprising a lecturer of Banking and Finance, a hospital client, and two hospital cashiers completed the face and content validation of the questionnaire through the Delphi method of information exchanges [24]. The questionnaire was pilot-tested among 30 managerial staff of privately owned hospitals. Concerns raised by the pilot participants were all fixed. The internal consistency (Cronbach’s α) score for each domain was: B = 0.75, C = 0.74, D = 0.78, E = 0.84, and F = 0.77. Sections A and G were considered nominal variables.

### Scoring

Section A was scored on a numerical scale of 0 to 5, representing the number of e-payment platforms in use. Sections B, C, D, E, and F were assessed on a 5-point Likert scale: strongly agree = 5, agree = 4, undecided = 3, disagree = 2, and strongly disagree = 1. The scores of each section were summed and converted to percentages for easy interpretability [25, 26]. Section G was demographic characteristics obtained as nominal or continuous variables.

### Procedure for data collection

We issued each participant a copy of the questionnaire for self-administration at their respective offices. We waited for them to complete and return the survey before moving to other office blocks. Participants needing further explanations or assistance were addressed on the spot. This procedure ensured high questionnaire completion and return rates.

### Variables

Continuous variables were participants’ age (years), the hospital’s e-payment usage (number of modes of payment), and the cumulative percentage scores for sections B to F. Gender (female = 0, male = 1), marital status (never married = 0, married/divorced/widowed = 1), and education (bachelorette = 0, postgraduate = 1) were nominal variables. The primary outcome was managerial disposition – defined by the participant’s attitude towards e-payment acceptance [17]. For example, participants were asked their level of agreement on the notion that e-payment would improve service delivery in the hospital. Perceived usefulness was defined as the degree to which participants believe e-payment enhances patients’ transaction experiences [27]. For instance, they were asked about the extent they believe e-payment would hasten transactions. Perceived ease of use was defined as participants’ belief in e-payment user-friendliness [27]. For example, they were asked how difficult they thought e-payment was. Credibility is defined as participants’ perception of the robustness and security of the e-payment platforms [19, 28]. They were asked how secure they thought e-payment was. Technophobia is participants’ fear of using e-payment platforms [29]. For example, they were asked the extent of their concerns about the potential e-payment failure.

### Data analysis

Data analysis was done using IBM SPSS Statistics, Version 22. The pilot-tested survey was analysed using Cronbach’s alpha. Descriptive statistics – mean, standard deviation, frequency, and percentage were used to summarise the respondents’ sociodemographic characteristics. The data had no missing variables, univariate outliers (standardised z-score ≥ ± 3.29) and multivariate outliers (Mahalanobis distance < 27.9). All the continuous variables except the hospital’s e-payment usage (number of platforms) met the assumption of normality (Shapiro-Wilk test), linearity (scatter plots), multicollinearity (correlation matrix), and Levene’s homoscedasticity test [30]. Hence, bivariate correlations among perceived usefulness, ease of use, credibility, technophobia, disposition, and the number of hospitals’ e-payment platforms were completed using Spearman’s correlation coefficient. Hierarchical multiple regression analysis was conducted to determine the influence of gender, marital status, educational level, age, perceived usefulness, perceived ease of use, perceived credibility, technophobia, and the number of e-payment platforms on the staff disposition.

## Results

**Fig. 1:**
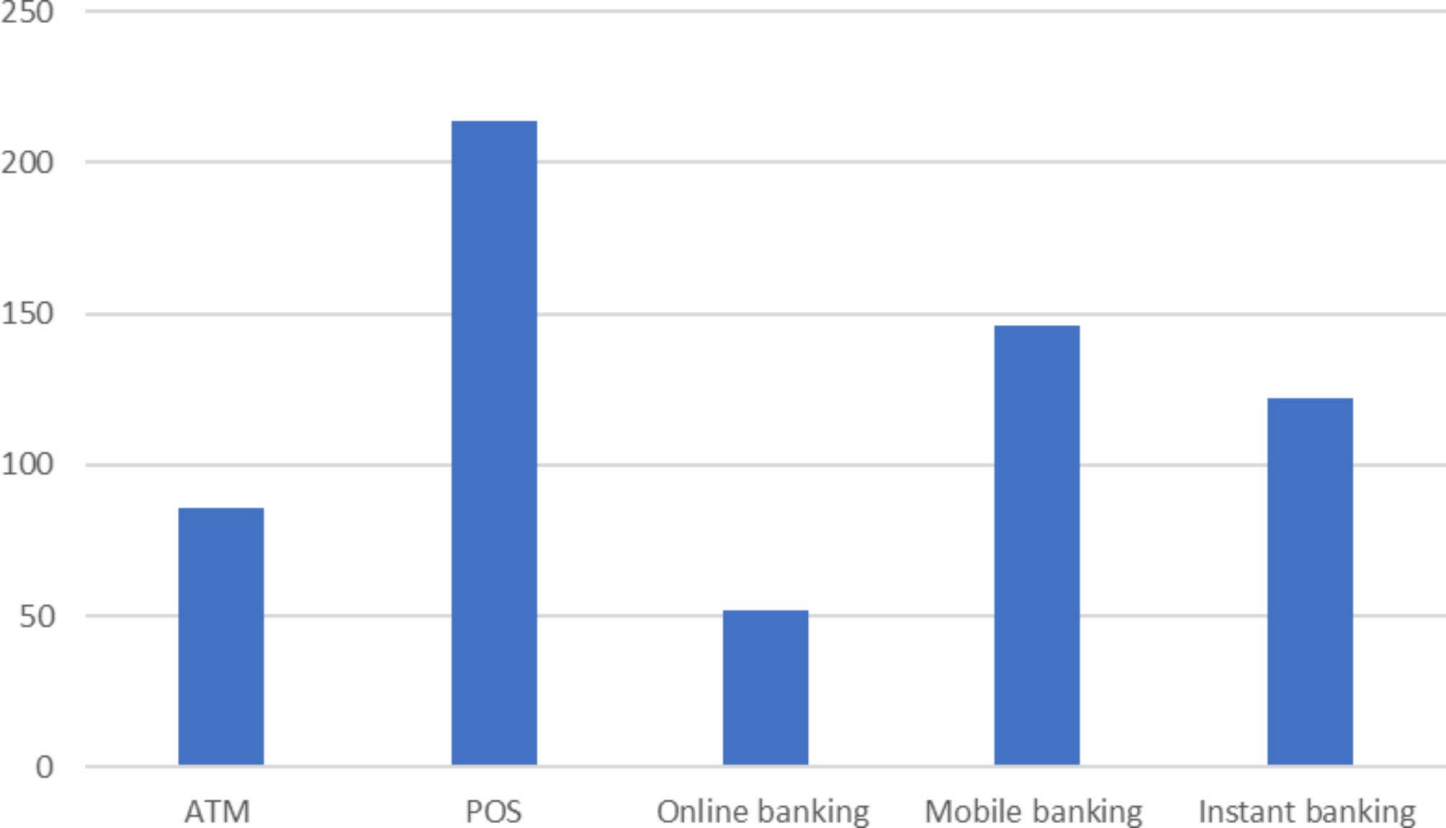
Distribution of respondent-reported e-payment usage across the hospitals

A total of 300 questionnaires were distributed and returned. However, 22 incomplete questionnaires were discarded, leaving 278 valid questionnaires in the analysis (92.7% completion rate). The participants’ sociodemographic data are displayed in Table 1. The majority of the respondents were married/divorced/widowed (n = 219, 78.8%), females (n = 164, 59%), between 36 and 45 years (n = 147, 52.9%), who had a bachelor’s degree (n = 207, 74.5%). The managerial staff’s mean ± standard deviation percentages of perceptions on the usefulness (85.7 ± 13.9%), and ease (80.5 ± 18.1%) of e-payment in the hospital were very positive, and credibility (72.6 ± 20.1%), and technophobia (68.0 ± 20.7%), were moderate. Their disposition to the use of e-payment in the hospital setting was positive (80.0% ± 17.9%). The large standard deviation showed that some respondents were at the extreme quartile of the distribution. Most respondents (n = 214, 77.0%) reported using up to three e-payment platforms in their hospitals. Figure 1 showed that the POS machine was the most common e-payment platform across the hospitals.

**Table 1:** Sociodemographic characteristics of the respondents

Variables	N (%)
**Age (years)**	
26–35	37 (13.3)
36–45	147 (52.9)
46–60	94 (33.8)
**Gender**	
Female	164 (59.0)
Male	114 (41.0)
**Marital status**	
Never married	59 (21.2)
Married/ Divorced / Widowed	219 (78.8)
**Educational level**	
Bachelors (BSc/BA/HND)	207 (74.5)
Postgraduate	71 (25.5)

Pearson’s correlation coefficients (Table 2) showed a significant positive relationship between managerial staff disposition and perceived usefulness (*ρ* = 0.63, *P* < 0.001), perceived ease of use (*ρ* = 0.60, *P* < 0.001), perceived credibility (*ρ* = 0.63, *P* < 0.001), and the number of e-payment platforms they believed were in use in their hospitals (*ρ* = 0.19, *P* = 0.001). However, managerial staff with higher technophobia had a higher negative disposition towards the use of e-payment in their hospitals (*ρ* = -0.50, *P* < 0.001).

**Table 2:** Correlations among staff perceptions and disposition to the use of e-banking in their hospitals

Variable	Perceived ease	Perceived credibility	Technophobia	E-payment platforms	Staff disposition
**Perceived usefulness**	*ρ* = 0.61 *P* < 0.001*	*ρ* = 0.49 *P* < 0.001*	*ρ* = -0.45 *P* < 0.001*	*ρ* = 0.14 *P* = 0.010*	*ρ* = 0.63 *P* < 0.001*
**Perceived ease**		*ρ* = 0.53 *P* < 0.001*	*ρ* = -0.44 *P* < 0.001*	*ρ* = 0.14 *P* = 0.008*	*ρ* = 0.60 *P* < 0.001*
**Perceived credibility**			*ρ* = -0.71 *P* < 0.001*	*ρ* = 0.18 *P* = 0.001*	*ρ* = 0.63 *P* < 0.001*
**Technophobia**				*ρ* = -0.17 *P* = 0.037*	*ρ* = -0.50 *P* < 0.001*
**Perceived number of e-payment platforms**					*ρ* = 0.19 *P* = 0.001*

*Significant at *P* < 0.05 (2-tailed)

Table 3 shows hierarchical multiple regression results. The first model was fitted using sociodemographic variables. In that order, the subsequent models (steps) were fitted by controlling for perceived usefulness, perceived ease of use, credibility, technophobia, and the number of e-payment platforms used by respondents’ hospitals. The best model was obtained in step 5 when technophobia was controlled for. The total variance explained (adjusted R^2^) was 12.9%, and the significant predictors of managerial disposition on e-payment were gender (β = -0.12, *P* = 0.033), marital status (β = 0.18, *P* = 0.003), and perceived credibility (β = 0.16, *P* = 0.05). Being a woman, married, and having a higher perception of credibility increases the propensity of managerial staff to have a more positive disposition towards the use of e-payment in the hospital.

**Table 3:** Hierarchical multiple regression: determinants of managerial staff disposition to the use of e-banking in their hospitals

	STEP 1		STEP 2		STEP 3		STEP 4		STEP 5	
Variable	β	*P*-value	β	*P*-value	β	*P*-value	β	*P*-value	β	*P*-value
Gender	-0.09	0.131	-0.10	0.075	-0.11	0.054	-0.13	0.032*	-0.12	0.033*
Marital status	0.17	0.005*	0.18	0.002*	0.18	0.003*	0.18	0.003*	0.18	0.003*
Education	0.14	0.062	0.10	0.177	0.10	0.197	0.09	0.219	0.09	0.223
Age	0.11	0.160	0.11	0.136	0.12	0.119	0.13	0.092	0.13	0.100
Perceived usefulness			0.14	0.025*	0.07	0.331	0.03	0.669	0.04	0.647
Perceived ease					0.11	0.145	0.05	0.494	0.05	0.492
Perceived credibility							0.15	0.032*	0.16	0.050*
Technophobia									-0.22	-0.821

*Significant at *P* < 0.05. β = Standardized coefficients. Step 1: gender, marital status, educational level, and age; Step 2: perceived usefulness; Step 3: perceived ease; Step 4: perceived credibility; Step 5: technophobia

Model summary

Step 1: *F* (4, 273) = 7.28, *P* < 0.001, *R* = 0.310, *R*^*2*^ = 0.096, *∆R*^*2*^ = 0.096, Adjusted *R*^*2*^ = 0.083

Step 2: *F* (5, 272) = 6.93, *P* < 0.001, *R* = 0.336, *R*^*2*^ = 0.113, *∆R*^*2*^ = 0.017, Adjusted *R*^*2*^ = 0.097

Step 3: *F* (6, 271) = 6.15, *P* < 0.001, *R* = 0.346, *R*^*2*^ = 0.120, *∆R*^*2*^ = 0.007, Adjusted *R*^*2*^ = 0.100

Step 4: *F* (7, 270) = 6.01, *P* < 0.001, *R* = 0.367, *R*^*2*^ = 0.135, *∆R*^*2*^ = 0.015, Adjusted *R*^*2*^ = 0.112

Step 5: *F* (8, 269) = 5.25, *P* < 0.001, *R* = 0.387, *R*^*2*^ = 0.155, *∆R*^*2*^ = 0.020, Adjusted *R*^*2*^ = 0.129

## Discussion

In this study, we explored the disposition of the managerial staff of public tertiary hospitals in Nigeria on e-payment in their hospitals. We also examined potential correlates of the managerial staff’s disposition towards e-payment, such as their perceptions of the benefits, ease of use, credibility, and technophobia towards e-payment platforms. Most respondents reported that three out of five common e-payment platforms are currently available in their hospitals. Specifically, POS, mobile banking, and instant banking were the most common. The overall disposition to the use of e-payment in the hospital was positive and statistically correlated with the number of e-payment platforms in respondents’ hospitals, perceived benefits, ease of use, and credibility of platforms. In contrast, there was a negative correlation between managerial staff’s disposition to e-payment and technophobia. Technophobia is fear and distrust of technology or the digitalisation of services. Furthermore, the multivariate analysis showed that gender and marital status were the significant sociodemographic determinants.

**Fig. 2:**
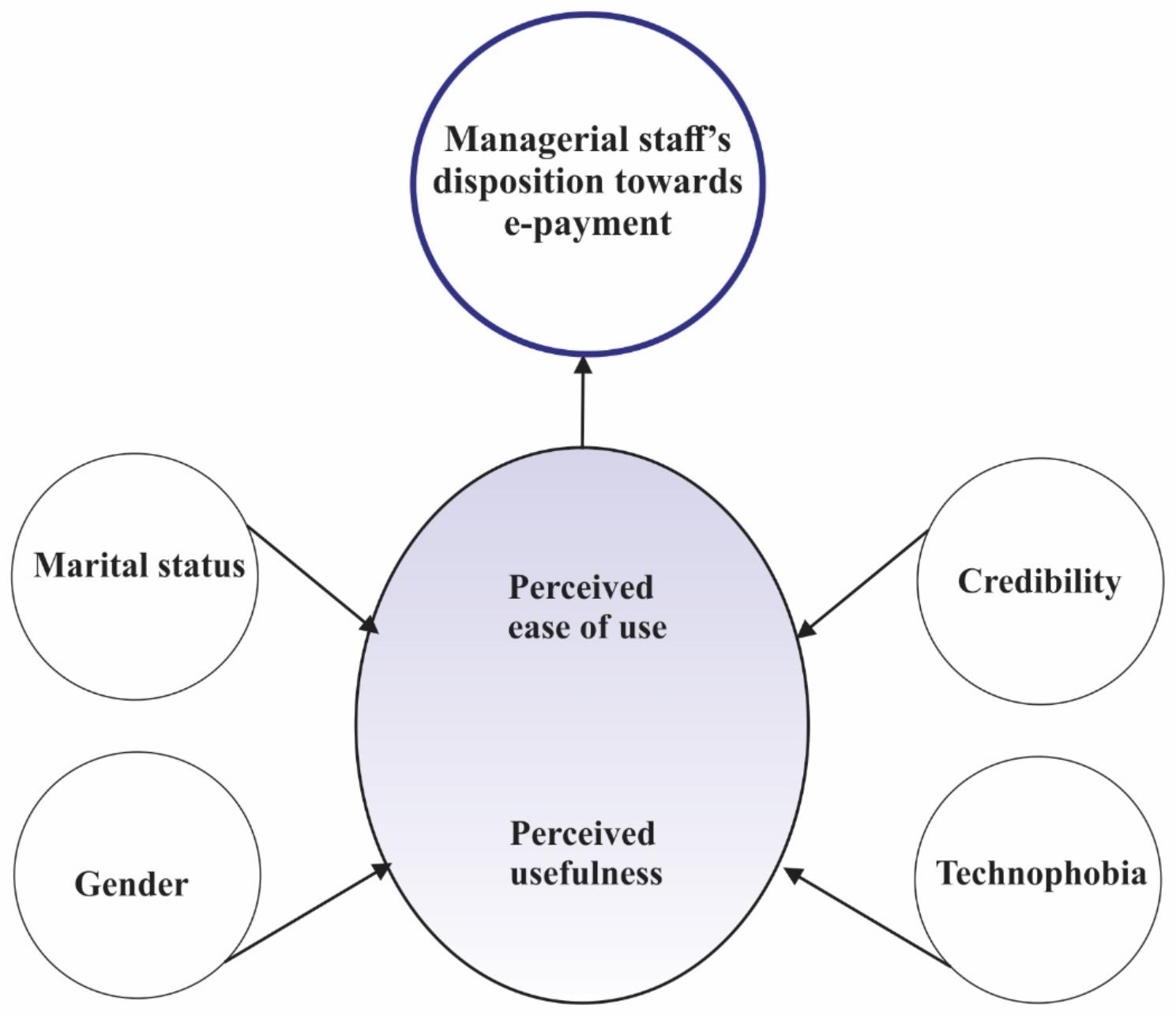
Framework for hospital managers’ e-payment acceptance in Enugu, Nigeria

From our findings, we developed a conceptual framework for hospital managers’ e-payment acceptance in Enugu, Nigeria (Fig. 2). Our model adapts and broadens TAM to the hospital setting within our socioeconomic and cultural context. Davis [18] proposed that the perception of use and usefulness mediated by system characteristics explains the potential of system acceptance. We further identified other correlates of managers’ disposition to the use of e-payment, including demographic factors, technophobia, and system characteristics such as credibility [19, 31]. Being a woman and married/divorced/widowed increased the managerial staff’s positive disposition to e-payment. In our context, women usually play the caregiving role to sick members of the family [32]. They dominate the formal healthcare workforce [5], and occupy more positions in the financial units of the hospital, exposing them to personal and clients’ experiences with the cash payment system.

Among the four psychological concepts we tested: perceived usefulness, ease of use, credibility and technophobia, our bivariate and multivariate analyses showed that perceived credibility was the most significant correlate of the managerial disposition towards e-payment. The more credible hospital management perceived the platform, the more positive their disposition to its use. This finding was consistent with previous studies that identified perceived credibility as a usage intention determinant [31, 33, 34]. Therefore, protecting hospitals against security threats such as fake payment alerts and hacking into the hospital’s e-banking account may improve the acceptability of the e-payment platforms. Ololade et al. [16] reported that the possibility of network intersection, issuance of fraudulent receipt of payment, and hacking of the receiving accounts the credibility of the e-payment systems. Similarly, Hertzum et al. [19] suggested upgrading e-payment user-interphase and security features to improve credibility.

Furthermore, the findings of this study were consistent with previous literature that found a positive correlation between the use of e-payment and perceived usefulness [8, 31, 35, 36]. Oni et al. [28] found that perceived usefulness is crucial in shaping a customer’s attitude and intention to use technology. This suggests that hospital managers’ receptiveness to e-payment may improve when they perceive them to be useful. Hence, demonstrating how e-payment platforms can address the delays in hospital transaction time may help facilitate their adoption and utility. Therefore, it is incumbent upon banks to provide adequate education to the bureaucrats in public hospitals to ensure they understand the value of e-payment platforms and address valuability concerns.

The Pearson coefficient showed that perceived ease of use positively affected managers’ disposition towards e-payment. However, this patient-centred concept was not significant in the multivariate models like those other concepts that directly affect hospital management, such as credibility and perceived usefulness. Previous studies on service consumers postulated that perceived ease or user-friendliness was a significant determinant of technology acceptance, as construed in TAM [17, 18, 27].

Technophobia negatively affected the managerial staff’s disposition towards using e-payment in hospitals. Sinkovics et al. [29] stated that necessity and convenience are crucial factors in technophobia. Gilbert et al. [37] found that technology anxiety is associated with demographic variables such as age, gender, and occupation, which can provide insight into consumers’ psychological and attributional factors. Bureaucrats are careful in collecting government revenue because all transactions are subjected to a third-party auditor who may not accept the failure of technology as an excuse for incomplete remittance. Beyond individual bills, the entire hospital’s account can be compromised by internet fraudsters [16]. The findings indicated that the participants perceived e-payment systems as helpful, convenient, and user-friendly. However, limitations in the security measures of e-payment technology and inadequate supporting facilities such as internet and electricity may deepen the technophobia among Nigerian service providers [13]. We recommend an interagency engagement between public hospital management, representatives of the supervisory government ministries, the banks, network security and software experts to develop an efficient and credible e-payment solution to ameliorate the plight of health services consumers in Nigeria. This study can catalyse inquiry into the hospital e-payment adoption across countries with similar health care procurement model and cash culture, especially in Sub-Saharan Africa.

### Strengths and limitations

While a few studies have investigated the customers’ perception of e-banking systems in Nigeria, this appears to be the first study on the policymakers’ disposition towards the use of e-payment for services delivered in Nigerian hospital settings. The sampling technique and sample size were sufficient, and face-to-face survey administration improved the questionnaire response and completion rates (92.7%).

This study has some limitations. Although we started with proportionate quota sampling across the hospitals, the within-hospital selection was done by consecutive sampling. The use of non-probability sampling may reduce the generalisability of our study. We would have collected the number of e-payment modes as administrative data across the hospitals from the management rather than relying on the reports from individual participants. The structured questionnaire survey may limit the respondents in expressing all their concerns and dispositions towards using e-payment in hospital settings.

Moreover, there could be social desirability bias when we asked managerial staff about their disposition towards e-payment. They may want to appear tech-savvy and compliant with the evolving National cashless policy. The study assumed that the clients desire the e-payment over the cash system and that they are tech-savvy to complete successful e-payment. However, there may be differences in financial culture across Nigeria’s sociopolitical regions, and cash preference still dominates some cultures.

## Conclusion

Electronic payment platforms are available in public tertiary hospitals in Enugu, Nigeria. The managers’ perceptions of usefulness, ease of use, and disposition towards e-payment were positive. However, perception of credibility and technophobia were moderate and significantly influenced managerial disposition. Demographic factors, such as being a woman and married, positively influenced the managerial staff’s disposition towards e-payment in the hospital. The bureaucrats, platform providers, and other stakeholders should collaborate to solve the problems of e-payment credibility and educate hospital management to reduce technophobia and improve the healthcare experiences of clients.

## Data Availability

The dataset for this study is available from the corresponding authors on reasonable request.

## Abbreviations

ATM: Automated teller machine
ESUTH: Enugu State University Teaching Hospital
FNHE: Federal Neuropsychiatric Hospital, Enugu
NHIS: National Health Insurance Scheme
NOHE: National Orthopedic Hospital, Enugu
STROBE: Strengthening the Reporting of Observational Studies in Epidemiology
TAM: Technology acceptance model
UNTH: University of Nigeria Teaching Hospital
